# Supplementary material for: Conveniently Pre-Tagged and Pre-Packaged: Extended Molecular Identification and Metagenomics Using Complete Metazoan Mitochondrial Genomes
Source: PLoS One. 2012 Dec 14;7(12):e51263. doi: 10.1371/journal.pone.0051263 (PMC3522660; doi:10.1371/journal.pone.0051263)
Supplement: File S2 — Sliding window analyses for Annelida, Cnidaria, Crustacea, Echinodermata, Mollusca, Nematoda, Nemertea, Platyhelminthes and Porifera. For each family, the folder contains the aligned sequences as well as the sliding window analyses by species pair and for all species pair on a single figure. (ZIP) [file pone.0051263.s002.zip › Platyhelminthes/Taeniidae/15_150/Echinococcus_shiquicus_NC_009460_Echinococcus_granulosus_NC_008075.pdf]

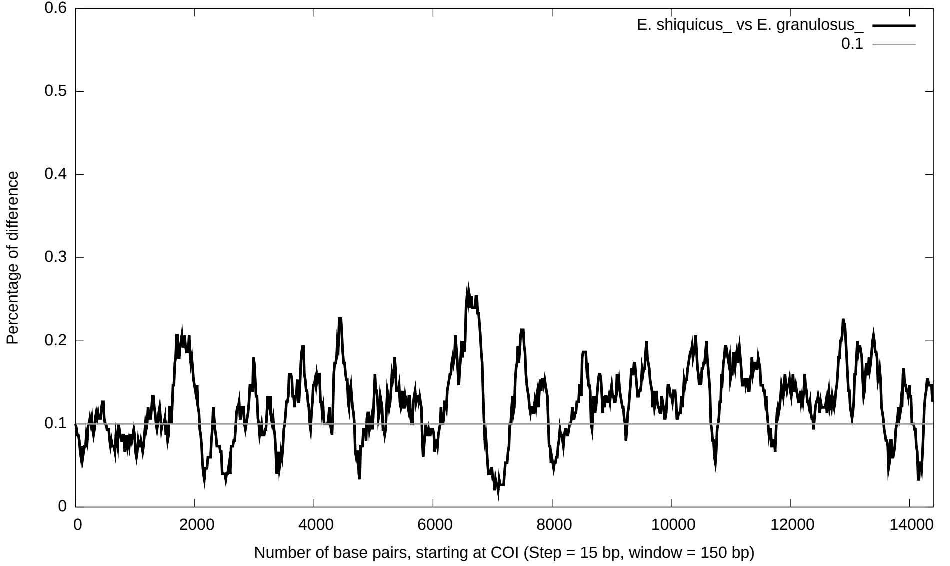

E. shiquicus_ vs E. granulosus_
0.1
0.6
0.5
0.4
0.3
0.2
0.1
0
Percentage of difference
0
2000
4000
6000
8000
10000
12000
14000
Number of base pairs, starting at COI (Step = 15 bp, window = 150 bp)
